# Individualized dual antiplatelet therapy based on platelet function testing in patients undergoing percutaneous coronary intervention: a meta-analysis of randomized controlled trials

**DOI:** 10.1186/s12872-017-0582-6

**Published:** 2017-06-15

**Authors:** Yijiang Zhou, Yanwei Wang, Yutao Wu, Chaoyang Huang, Hui Yan, Weiguo Zhu, Weiwei Xu, Li Zhang, Jianhua Zhu

**Affiliations:** 1 Department of Cardiology, The First Affiliated Hospital, Zhejiang University, School of Medicine, 79 Qingchun Road, Zhejiang, Hangzhou 310003 China; 2 Department of Cardiology, Ningbo Medical Treatment Center Lihuili Hospital, Ningbo, 315000 China; 3 Department of Endocrinology, The First Affiliated Hospital, Zhejiang University, School of Medicine, 79 Qingchun Road, Hangzhou, 310003 China

**Keywords:** Clopidogrel, Dual antiplatelet therapy, Percutaneous coronary intervention, High on-treatment atelet reactivity

## Abstract

**Background:**

High on-treatment platelet reactivity (HPR) represents a strong risk factor for thrombotic events after PCI. We aim to evaluate the efficacy and safety of individualizing intensified dual antiplatelet therapy (DAPT) in PCI-treated patients with HPR based on platelet function testing (PFT).

**Methods:**

Electronic databases were searched for randomized control trials that reported the clinical outcomes of using an intensified antiplatelet protocol with P2Y_12_ receptor inhibitor comparing with standard maintenance dose of clopidogrel on the basis of platelet function testing. Clinical endpoints were assessed.

**Results:**

From 2005 to 2016, thirteen clinical studies comprising 7290 patients were included for analysis. Compared with standard antiplatelet therapy with clopidogrel, the intensified protocol based on platelet function testing was associated with a significant reduction in major adverse cardiovascular events (RR:0.55, 95% CI: 0.36–0.84, *p* = 0.005), cardiovascular death (RR:0.60, 95% CI: 0.38–0.96, *p* = 0.03), stent thrombosis (RR:0.58, 95% CI: 0.36–0.93, *p* = 0.02) and target vessel revascularization (RR:0.33, 95% CI: 0.14–0.76, *p* = 0.009). No significant difference was found in the rate of bleeding events between intensified and standard protocol.

**Conclusions:**

Compared with standard clopidogrel therapy, individualized intensified antiplatelet therapy on the basis of platelet reactivity testing reduces the incidence of cardiovascular events in patient undergoing PCI, without increasing the risk of bleeding.

**Electronic supplementary material:**

The online version of this article (doi:10.1186/s12872-017-0582-6) contains supplementary material, which is available to authorized users.

## Background

Dual antiplatelet therapy (DAPT) with aspirin and a P2Y_12_ antagonist represents the standard of care in patients with acute coronary syndrome (ACS) and those undergoing percutaneous coronary interventions (PCI) [1]. However, a concerning issue is that wide inter-individual variability exists among P2Y_12_ antagonist, especially clopidogrel [2–4]. The incidence of high on-treatment platelet reactivity (HPR) can be as high as in one-third of patients treated with clopidogrel, and HPR is present even in those receiving the more potent P2Y_12_ antagonists ticagrelor and prasugrel [5]. Numerous studies have demonstrated that HPR is associated with increased cardiovascular death and thrombotic events [6, 7]. Several small clinical studies have shown that increasing the dose of clopidogrel or switching to a more potent P2Y_12_ receptor inhibitor in patients with HPR can significantly reduce the incidence of major adverse cardiac events (MACE) [8–10]. However, large randomized trials failed to demonstrate similar improvements in clinical outcomes with a tailored antiplatelet therapy based on platelet function monitoring [11–14]. Therefore, though some studies found platelet reactivity-guided antiplatelet therapy to be beneficial in certain clinical endpoints like bleeding and stent thrombosis [15], the usefulness of tailored antiplatelet therapy during PCI is still controversial. In the present study, we performed meta-analysis on previous studies to evaluate the clinical efficacy and safety of individualized intensification of antiplatelet therapy with P2Y_12_ inhibitors versus standard dose clopidogrel on the basis of platelet function testing(PFT) in patients undergoing PCI.

## Methods

PubMed, MEDLINE and ClinicalTrials were searched for clinical studies published between January 2005 and September 2016. The key words we used included the following terms: ‘platelet function’, ‘platelet reactivity’, ‘platelet aggregation’, ‘platelet aggregometry’, ‘antiplatelet resistance’,‘high on treatment platelet reactivity’,‘P2Y_12_-ADP receptor inhibitor’, ‘clopidogrel’, ‘prasugrel’, ‘ticagrelor’ and ‘percutaneous coronary intervention’. In addition, references of relevant studies and reviews, editorials, and letters, together with related conference abstracts were also searched.

The main criteria for inclusion in this analysis were trials that compared clinical efficacy and/or safety of an intensified antiplatelet therapy with that of standard-dose clopidogrel therapy on the basis of platelet reactivity testing in patients undergoing PCI. Studies without platelet function testing were excluded. Similarly, studies that compared only the pharmacological efficacy of a platelet function-guided anti-platelet therapy were also excluded. Moreover, studies in which platelet inhibitor other than P2Y_12_ inhibitors were used were excluded.

According to international consensus and previous studies [16, 17],the accepted ADP-specific platelet function devices were: (a) VerifyNow P2Y_12_ assay, (b) Multiplate analyzer with ADP test, (c) Flow cytometric assessment of vasodilator stimulated phosphoprotein (VASP) phosphorylation index, and (d) Conventional light transmission aggregometry (LTA).

The efficacy endpoints of the analysis included: (a) Major adverse cardiovascular events (MACE), (b) Cardiovascular death, (c) Definite/probable ST, (d) Myocardial infarction (MI), (e) Stroke and (f) Target vessel revascularization (TVR). All of them were defined according to the study definition. In particular, MACE was primarily defined as a composite of cardiovascular death, myocardial infarction and definite/probable stent thrombosis, though a few studies also included endpoint of stroke or transient ischemic attack (TIA). The main safety endpoint was major bleeding and major or minor bleeding events, which were defined according to the study definition,including BARC, TIMI, GUSTO and STEEPLE [18–21]. The net clinical end point was defined as a composite of MACE and major bleeding.

Two investigators independently assessed reports for eligibility on the title and/or at abstract level, with divergences resolved with a third reviewer; studies that met inclusion criteria were selected for further analysis. The risk of bias was evaluated by the same two reviewer authors, in accordance with The Cochrane Collaboration methods [22].

Meta-analysis was performed using the Review Manager 5.3 and STATA 14 statistical software. Reported event frequencies were used to calculate risk ratios (RR) with 95% confidence intervals (CI). Heterogeneity of the trial results was quantified with the Chi^2^ heterogeneity statistic, with inconsistency assessed by means of I^2^. Results were reported as the *p* value of the Chi^2^ test (*p* < 0.05 for heterogeneous results) and percentage of the I^2^. Interpretation of the latter was made by assigning attributes of low, moderate, and high in case of 0–25%, 50–75% and more than 75%, respectively. We used a random-effects or a fixed-effect model based on degree of heterogeneity. The random-effects model results in wider confidence intervals and provides more conservative and robust results, and it was used when I^2^ > 50%. Subgroup analyses were performed according to strategies to overcome HPR, intervention duration, follow-up duration and method of platelet function testing. Analysis of variance(ANOVA) were further carried out between different subgroups. To determine the impact of baseline risks and study characteristics on the MACE and net clinical benefit, meta-regression analyses were performed with STATA 14 software. To study the relevance of publication bias, funnel plots were constructed plotting the trial results against their precision. Begg’s regression intercept was used to assess asymmetry of these funnel plots. Only composite clinical endpoints were used from the meta-analysis. The Duval and Tweedie’s “Trim and Fill” method was used to impute ‘hypothetical’ missing studies and to calculate adjusted versus observed RRs.

## Results

**Fig. 1:**
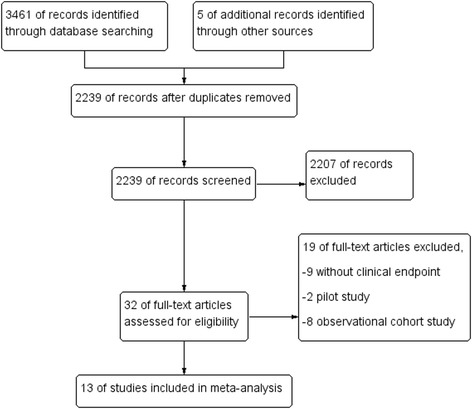
Flowchart of study selection

**Fig. 2:**
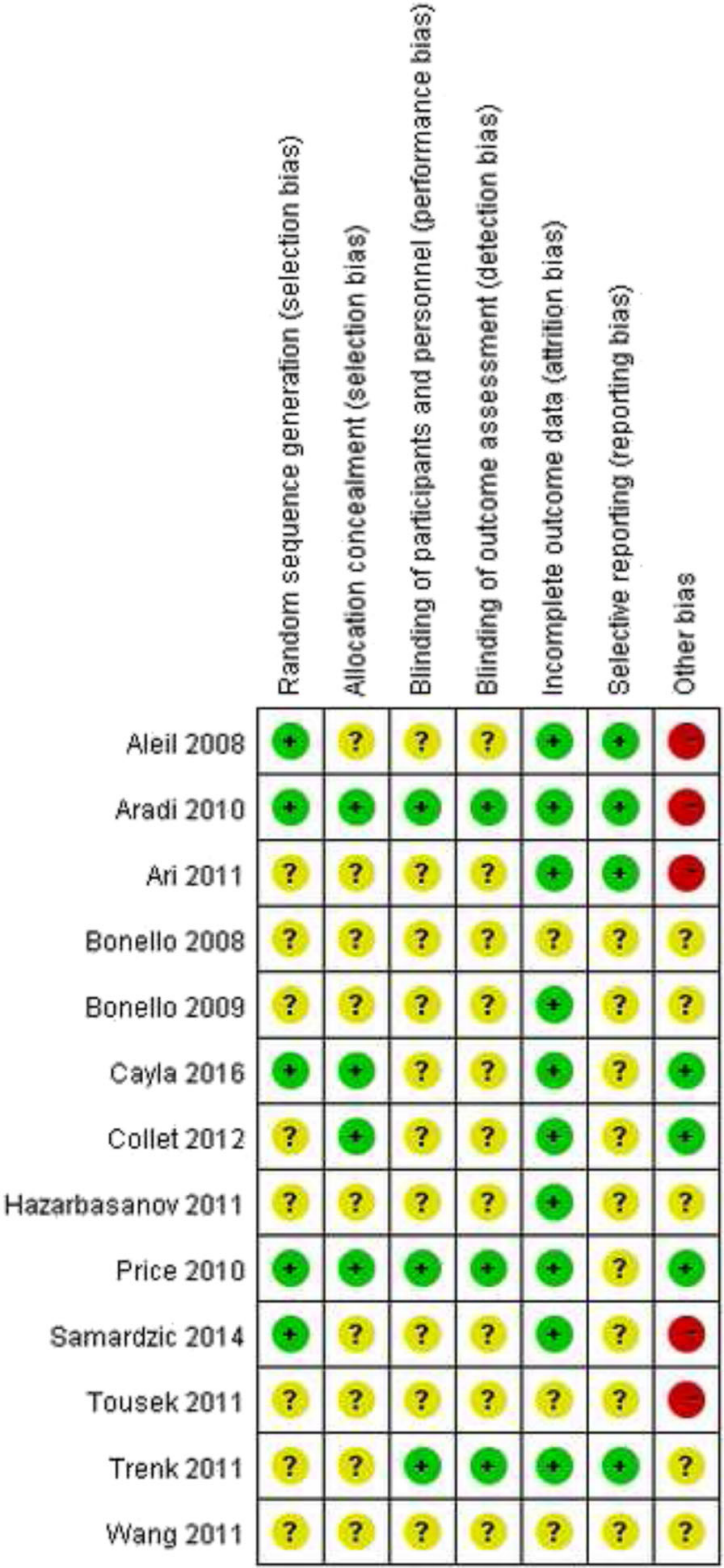
Summary table of review authors’ judgements for each risk of bias item for each study

Through searching with aforementioned key words, 3466 reports were retrieved, of which 2239 relevant publications were identified at the abstract and title level. Full text assessment was performed in 32 trials. By applying the inclusion and exclusion criteria, thirteen studies involving 7290 (range: 60–2440) patients were qualified for the analysis (Fig. 1). A summary table of review authors’ judgements for each risk of bias item for each study was shown in Fig. 2. The detailed characteristics of the included studies are shown in Table 1. Studies vary according to publication year, risk profiles of the recruited patients, platelet function assay used, definition of HPR, intensified antiplatelet strategy applied and clinical endpoints. All studies used P2Y_12_ antagonist for intensified antiplatelet regime: two studies administered repeated loading dose of 600 mg clopidogrel [8, 9]; seven studies increased maintenance dose of clopidogrel [11, 23–28]; two studies used repeated loading dose of 600 mg clopidogrel combined with increased maintenance dose of clopidogrel or prasugrel [29, 30]; another two studies used prasugrel for loading and maintenance [13, 14].

**Table 1:** Study characteristics

Author	Acronym	Date	Blinding	Patient no. (tailored/control)	Platelet function assay	HPR cut-off
Aleil		2008	No	31/62	VASP	VASP-PRI > 69%
Bonello		2008	No	78/84	VASP	VASP-PRI > 50%
Bonello		2009	No	215/214	VASP	VASP-PRI > 50%
Aradi	DOSER	2010	Yes	36/38	LTA, 5 μM ADP	>34%AGGmax
Price	GRAVITAS	2010	No	1109/1105	VerifyNow,P2Y12	>230PRU
Ari	EFFICIENT	2011	Yes	47/47	VerifyNow P2Y12	<40%inhibition
Hazarbasanov		2011	No	97/95	VerifyNow P2Y12	>208PRU
Tousek		2011	No	30/30	VerifyNow P2Y12	>240PRU
Trenk	TRIGGER-PCI	2011	Yes	212/211	VerifyNow P2Y12	>208PRU
Wang		2011	No	150/156	VASP	VASP-PRI > 50%
Collet	ARCTIC	2012	No	1213/1227	VerifyNow,P2Y12	>235PRU
Samardzic		2014	No	43/44	MEA	>46 U
Cayla	ANTARCTIC	2016	No	435/442	VerifyNow,P2Y12	>208PRU
Author	Clinical setting		Modified treatment		Intervention duration	Follow-up
Aleil	Scheduled for elective coronary stenting		150 mg MD clopidogrel		2 weeks	1 month
Bonello	Stable angina:52% NSTEMI:48% STEMI: 0%		Repeated 600 mg clopidogrel LD		One week	30 days
Bonello	Stable angina:48% NSTEMI:52% STEMI: 0%		Repeated 600 mg clopidogrel LD		One week	30 days
Aradi	Stable angina:100%		600 mg LD +150 mg MD clopidogrel		1 month	12 months
Price	Stable angina:60% NSTEMI:10.1% STEMI: 0.4%		600 mg LD +150 mg MD clopidogrel		6 months	6 months
Ari	Stable angina:100%		150 mg MD clopidogrel		1 month	6 months
Hazarbasanov	Stable angina:43% NSTEMI:33% STEMI: 24%		600 mg LD +150 mg clopidogrel		1 month	6 months
Tousek	Stable angina:23% NSTEMI:34% STEMI: 43%		clopidogrel starting at 150 mg/day with further dose increase		1 month	6 months
Trenk	Stable angina:100%		60 mg LD +10 mg MD Prasugrel		6 months	6 months
Wang	Stable angina:80% NSTEMI:20% STEMI: 0%		increase in clopidogrel MD up to 375 mg		11 months	1 year
Collet	Scheduled for elective coronary stenting		Repeated 600 mg clopidogrel LD + 150 mg MD clopidogrel/10 mg MD Prasugrel		12 months	12 months
Samardzic	Stable angina:16% NSTEMI:23%STEMI:60%		up to clopidogrel 600 mg × 2 + (75-300 mg MD)		12 months	12 months
Cayla	Stable angina:18% NSTEMI:48%STEMI:34%		5–10 mg MD Prasugrel		12 months	12 months

*Abbreviations ACS* acute coronary syndrome, *CV* cardiovascular, *HTPR* high on-treatment platelet reactivity, *LD* loading dose, *MD* maintenance dose, *MI* myocardial infarction, *STEMI* ST-segment elevation myocardial infarction, *NSTEMI* non-ST segment elevation myocardial infarction, *ST* stent thrombosis, *TVR* target vessel revascularization, *MACE* major adverse cardiovascular events *RCT* randomized, controlled trial

**Fig. 3:**
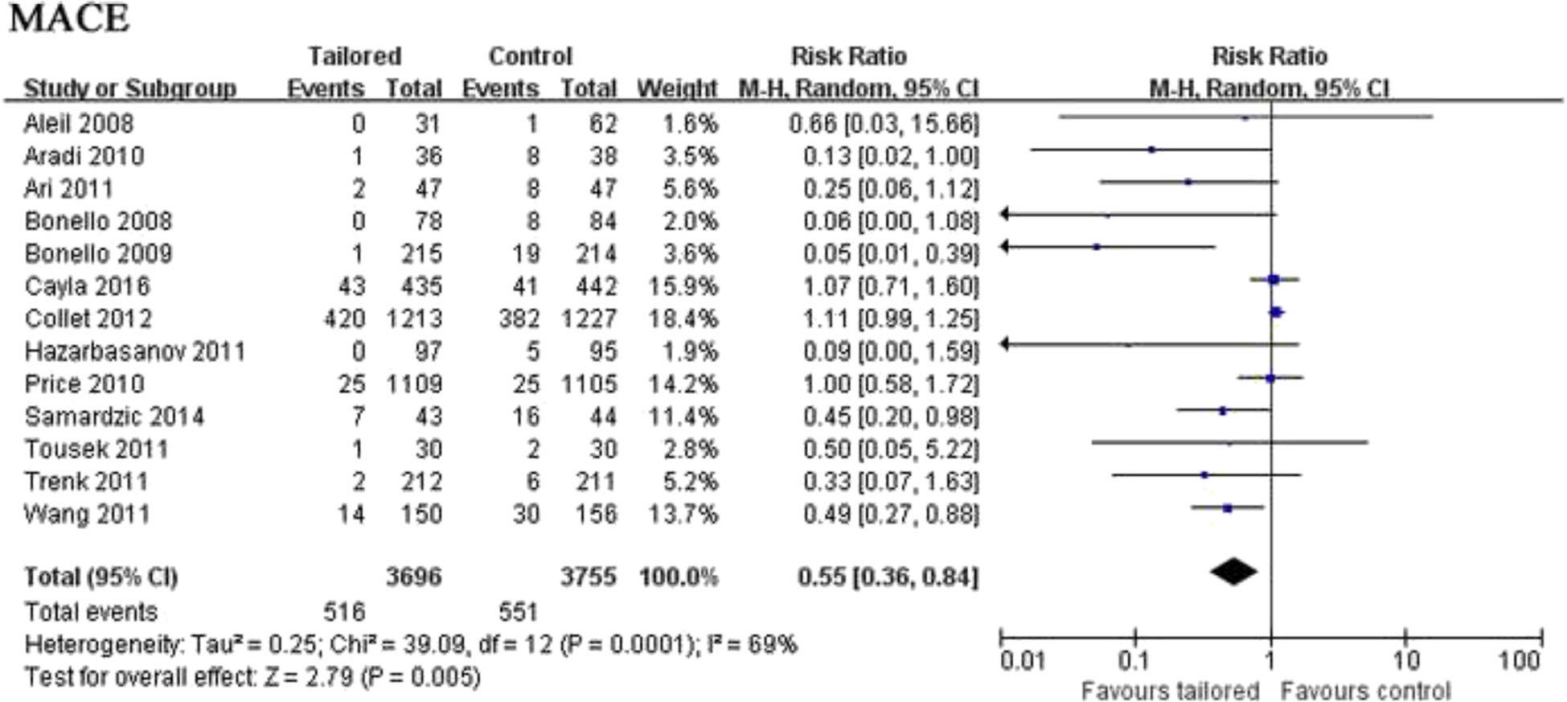
Forest plot for incidence of major adverse cardiovascular events. Risk ratio for individual studies (*squares*) and meta-analysis (*diamonds*) and 95% CI (*horizontal lines*) are presented

**Fig. 4:**
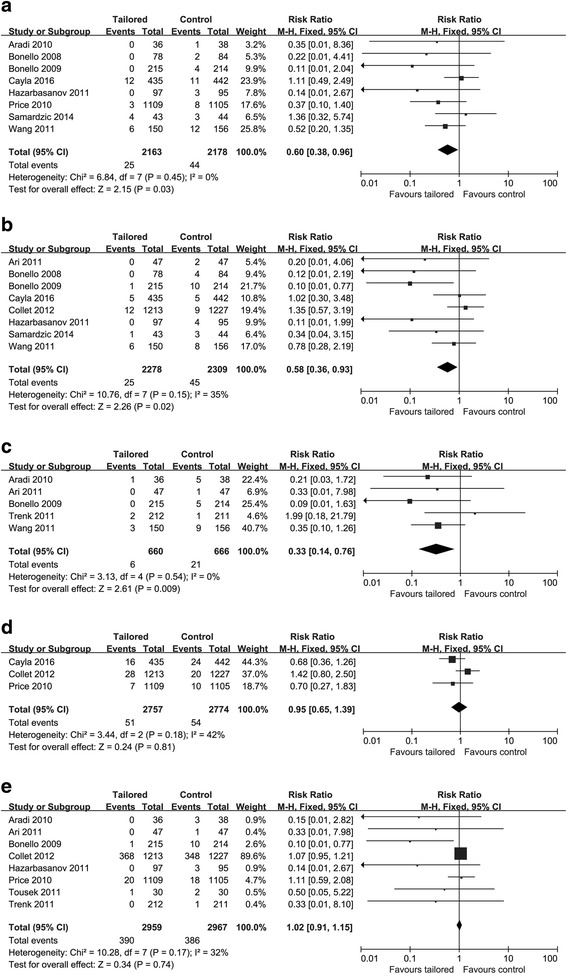
Forest plot for incidence of cardiovascular death, **a** stent thrombosis **b**, target vessel revascularization, **c** death **d** and myocardial infarction **e**. Risk ratio for individual studies (squares) and meta-analysis (diamonds) and 95% CI (horizontal lines) are presented

Based on the pooled results, the intensified therapy was associated with a significant reduction in major adverse cardiovascular events (MACE) (RR: 0.55, 95% CI: 0.36–0.84, *p* = 0.005; Fig. 3). Moreover, intensified antiplatelet strategy guided by platelet function testing reduced rate of cardiovascular death (RR: 0.60, 95% CI: 0.38–0.96, *p* = 0.03), definite/probable stent thrombosis (RR: 0.58, 95% CI: 0.36–0.93, *p* = 0.02) and target vessel revascularization (TVR) (RR: 0.33, 95% CI: 0.14–0.76, *p* = 0.009) (Figs. 4a-c). On the other hand, there was no difference in incidence of death from any cause (RR: 0.95, 95% CI: 0.65–1.39, *p* = 0.81) or myocardial infarction (RR: 1.02, 95% CI: 0.91–1.15, *p* = 0.74) between platelet function-guided intensified antiplatelet strategy with standard maintenance dose of clopidogrel therapy (Fig. 4d-e).

**Fig. 5:**
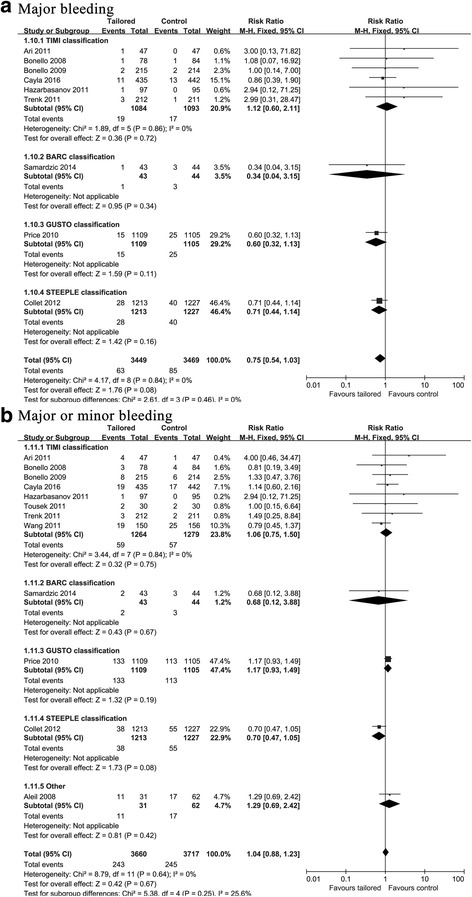
Forest plot for incidence of major bleeding **a** and major or minor bleeding **b**. Risk ratio for individual studies (squares) and meta-analysis (diamonds) and 95% CI (horizontal lines) are presented

No difference in the rate of major bleeding events (RR: 0.75, 95% CI: 0.54–1.03, *p* = 0.08) or major or minor bleeding events (RR: 1.04, 95% CI: 0.88–1.23, *p* = 0.67) was observed between the two therapeutic groups (Fig. 5).

**Fig. 6:**
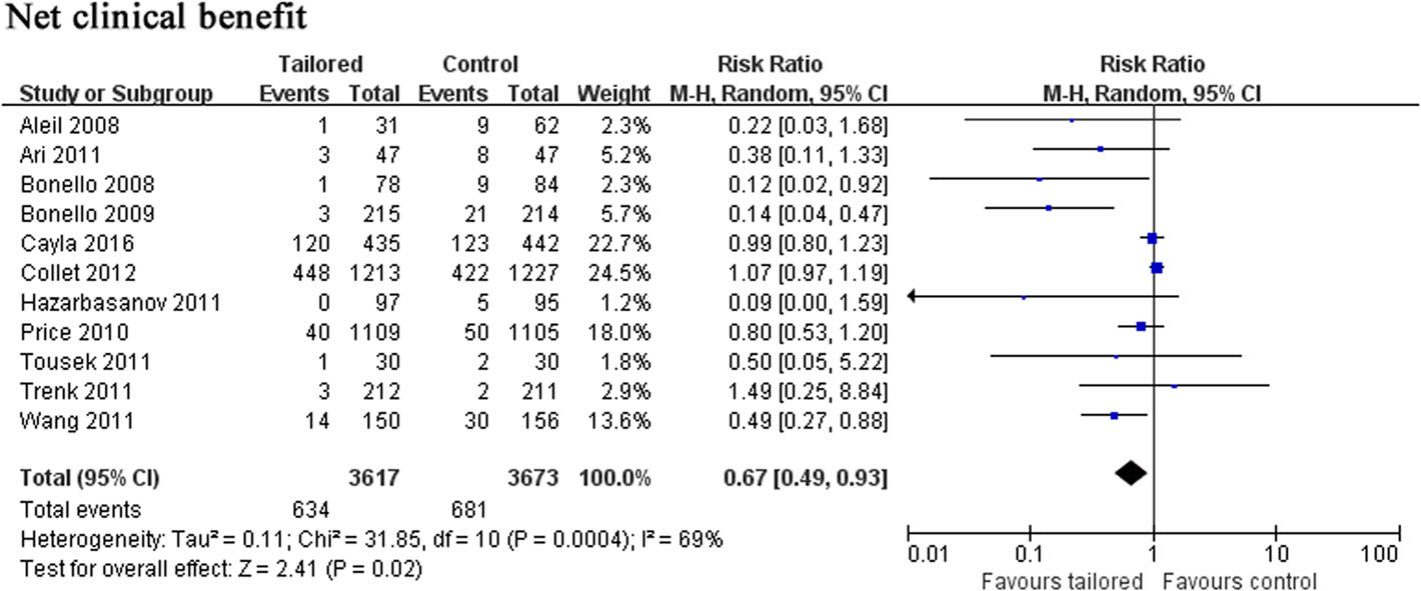
Forest plot for incidence of net clinical benefit. Risk ratio for individual studies (squares) and meta-analysis (diamonds) and 95% CI (horizontal lines) are presented

The net clinical benefit, consisting of both thrombotic and bleeding events, was also superior in intensified therapy (RR: 0.67, 95% CI: 0.49–0.93, *p* = 0.02) (Fig. 6).

Subgroup analyses were performed. According to strategies to overcome HPR (repeated clopidogrel loading, increased clopidogrel maintenance dose or switching to prasugrel), pooled results showed that repeated loading or increased maintenance dose of clopidogrel significantly reduced the incidence of MACE without increasing major bleeding events, favoring a net clinical benefit. However, switching to prasugrel was similar with standard therapy in MACE, major bleeding and net clinical benefit. Pooled results from trials with intervention duration of 1 month or less and follow-up time of 1 month showed that intensified antiplatelet strategy significantly decreased the MACE, cardiovascular death and net clinical events. Results from studies using VASP showed that intensified therapy was associated with less incidence of MACE, cardiovascular death and net clinical events (Table 2). ANOVA analyses were also performed between subgroups according to strategies to overcome HPR, intervention duration, follow-up duration and platelet function testing, showing that differences across subgroups in MACE and net clinical events are significant (Table 2). What’s more, pooled result from studies only included patients with stable coronary artery disease showed that intensified therapy decreased the incidence of MACE (RR: 0.23, 95%CI: 0.09–0.60, *p* = 0.002 (Additional file 1).

**Table 2:** Subgroup analysis

	MACE			Cardiovascular death			Major bleeding			Net clinical events		
	RR(95% CI)	I^2^ (%)	*p*	RR(95% CI)	I^2^ (%)	*p*	RR(95% CI)	I^2^ (%)	*p*	RR(95% CI)	I^2^ (%)	*p*
*Strategies to overcome HPR*			0.02			0.26			0.60			<0.001
Repeated LD	0.06(0.01–0.29)	0	<0.001	0.15(0.02–1.18)	0	0.07	1.02(0.21–5.02)	0	0.98	0.14(0.05–0.38)	0	<0.001
Increased MD	0.56(0.39–0.96)	66	0.003	0.41(0.20–0.84)	0	0.01	0.69(0.38–1.25)	0	0.22	0.59(0.44–0.81)	10	0.001
Switch to prasugrel	0.77(0.28–2.15)	49	0.62				1.01(0.49–2.11)	5	0.97	1.00(0.81–1.24)	0	1.00
*Intervention duration*			<0.001			0.06			0.26			<0.001
≤1 month	0.16(0.07–0.37)	0	<0.001	0.17(0.04–0.75)	0	0.02	1.51(0.43–5.34)	0	0.52	0.21(0.11–0.42)	0	<0.001
>1 month	0.81(0.58–1.14)	65	0.23	0.75(0.45–1.25)	6	0.27	0.71(0.51–0.99)	0	0.05	0.93(0.75–1.14)	54	0.47
*Follow up duration*			0.03			0.11			0.91			<0.001
1 month	0.09(0.02–0.40)	0	0.001	0.15(0.02–1.18)	0	0.07	1.02(0.21–5.02)	0	0.98	0.15(0.06–0.38)	0	<0.001
6 months	0.48(0.21–1.11)	38	0.09	0.30(0.09–1.00)	0	0.05	0.77(0.44–1.36)	11	0.38	0.72(0.49–1.08)	2	0.11
12 months	0.73(0.46–1.14)	75	0.16	0.84(0.49–1.15)	0	0.53	0.72(0.49–1.08)	0	0.11	0.93(0.72–1.21)	71	0.60
*Platelet function device*			0.05			0.27			0.72			<0.001
VerifyNow	0.94(0.70–1.27)	37	0.69	0.69(0.37–1.32)	39	0.26	0.76(0.54–1.05)	0	0.10	0.98(0.82–1.16)	26	0.79
VASP	0.20(0.05–0.91)	58	0.04	0.38(0.16–0.89)	0	0.03	1.02(0.21–5.02)	0	0.98	0.27(0.12–0.59)	38	0.001

To evaluate the impact of baseline clinical risks and study characteristics on the MACE and net clinical benefit of the individualized therapy, we performed meta-regression for different variables, including percentage of ACS patients, study size, patient age, cut-off value for HPR, follow-up time (≤1 months or >1 months) and intervention duration (1 month, 6 months or 12 months) (Table 3). Our analysis showed that study size and intervention duration were significantly associated MACE, and the above two factors as well as follow-up time were associated with net clinical benefit, suggesting the heterogeneity in clinical efficacy were partially explained by these factors .

**Table 3:** Meta-regression analyses for MACE and net clinical benefit

	MACE				Net clinical benefit			
	*p*	Adj-R^2^ (%)	I^2^ (%)	Tau^2^	*p*	Adj-R^2^ (%)	I^2^ (%)	Tau^2^
Percentage of ACS	0.028	7.59	54.65	0.3979	0.211	0	54.62	0.4356
Age	0.229	0	70.27	0.456	0.195	8.99	50.75	0.2937
Verifynow cutoff value	0.566	0	22.14	0	0.864	0	20.59	0.1935
Study size	0.005	44.13	46.13	0.2406	0.032	4.91	66.22	0.3069
Follow up	0.077	22.45	60.23	0.3181	0.022	61.72	40.97	0.1219
Intervention duration	0.005	76.06	36.87	0.09818	0.006	86.69	18.98	0.04239

To check for the possibility of a publication bias, we used Begg’s test and found no publication bias in cardiovascular death (*p* = 0.536), definite/probable ST (*p* = 0.108), myocardial infarction (*p* = 0.386), MACE (*p* = 0.583), major bleeding (*p* = 0.175) and net clinical benefits (*p* = 0.350). Furthermore, sensitivity analysis by Trim and Fill method showed that the pooled results of MACE and net clinical benefits were reliable.

## Discussion

The results our meta-analysis showed that in patients undergoing PCI with HPR, a strategy of individualized intensification of dual antiplatelet therapy reduces adverse cardiovascular events, including cardiovascular death, stent thrombosis and target vessel revascularization, without increasing hemorrhagic complications.

Dual antiplatelet therapy with aspirin and P2Y_12_ antagonists has become the standard therapy for preventing adverse cardiovascular events after PCI. However, considerable inter-individual variability exists among patients receiving clopidogrel. Many observational studies have established HPR as the risk factor for ischemic events after PCI, but whether modifying HPR with intensified antiplatelet regime can improve cardiovascular outcome is still controversial. AHA/ACC guidelines of PCI recommend that in patients at high risk of stent thrombosis, platelet aggregation test may be applied, and that if platelet inhibition is inadequate, clopidogrel dose may be increased [31, 32]. Since the prognostic value of bleeding complications is equally important as ischemic events, a personalized approach to tailor the antiplatelet therapy for patients with variable on-treatment platelet reactivity is an attractive strategy. Though small clinical trials have demonstrated that increasing the dose of clopidogrel in patients with HPR can reduce the incidence of major adverse cardiac events, some large randomized trials failed to demonstrate an improvements in clinical outcomes with a tailored anti-platelet therapy based on platelet-function monitoring [11, 13, 29]. The small number of events in low risk patients included in these trials might be accountable for negative results. Moreover, poor responsiveness is likely due to multifactorial reasons, including clinical, cellular and genetic factors, with gene polymorphisms emerging as one of the major determinants of clopidogrel response [33–35]. Different mutations may lead to discrepant gene function, therefore resulting in poor to no clopidogrel response. Intensified anti-platelet regime used in these trials might overcome certain types of mutations, but could not achieve sufficient platelet inhibition in patients with specific genotype. What’s more, recent studies demonstrated that the magnitude of the association between HPR on clopidogrel treatment and incidence of MACE is strongly dependent on the level of patients’ cardiovascular risk [36].

Previously published meta-analysis on intensified antiplatelet therapy on the basis of platelet reactivity testing suggested that in patients undergoing PCI who have HPR, more potent antiplatelet regime reduced the rate of thrombotic events without increasing the risk for bleeding events [37, 38]. But these studies synthesized different classes of antiplatelet agents, including peri-procedural use of glycoprotein IIb/IIIa receptor inhibitors (GPIs). Our study focused on dual antiplatelet therapy with aspirin and P2Y_12_ antagonists only, since we believe with broader application of newer P2Y_12_ inhibitors, procedural use of GPI would be gradually decreasing. Our results clearly showed that individualized intensification of dual anti-platelet therapy reduced adverse cardiovascular events,which might mainly derive from the decreasing of cardiovascular death, stent thrombosis and target vessel revascularization, leading to net clinical benefit.

From our subgroup analysis, we found strategy of overcoming HPR had a major impact on clinical efficacy. While repeated loading or increased maintenance dose of clopidogrel significantly reduced incidence of MACE and favored net clinical benefit, it is intriguing to find the newer P2Y_12_ inhibitors prasugrel did not. Though the reason remained unknown, possible explanations are: 1) number of studies that used prasugrel (only TRIGGER-PCI and ANTARCTIC study) is far less than those used higher doses of clopidogrel, potentially biasing the result; 2) TRIGGER-PCI study included patients at low ischemic rate, possibly masking the benefit of prasugrel; 3) ANTARCTIC study only enrolled patients of 75 years or older, its failure to demonstrate the benefit of prasugrel is limited to this sub-population. Whether PFT-based prasugrel dosing could reduce ischemic event in a broader patient population with higher risks needs further investigation. It would also be interesting test another new P2Y12 inhibitor, ticagrelor. Our analysis and meta-regression analysis also showed studies with short intervention duration (≤1 month) and follow-up time (1 month) were more beneficial than those with longer intervention or follow-up duration in the endpoints of MACE, cardiovascular death and net clinical events. It seems reasonable that the PFT-guided anti-platelet therapy is prone to reduce shorter-term cardiovascular events, since platelet function testing are mostly performed peri-procedurally and reflects peri-procedural thrombotic burden. It is also interesting from our analysis that considering studies using only Verifynow, such strategy did not reduce any cardiovascular outcomes, as opposed to VASP. One reason is that studies using Verifynow adopted different thresholds for HPR, ranging from >208 PRU to >240 PRU. Also possible is that VerifyNow-P2Y12 cartridge might be influenced by many factors such as time between process, platelet count, hematocrit and etc. However, whether VASP is indeed better than VerifyNow in guiding individualized DAPT needs further study.

Previous studies reported inconsistent results in the relationship between HPR and bleeding. Sibbing et al. showed that patients with and without HPR had similar incidence of major and minor bleeding [4]. However, ADAPT-DES trial found that HPR was inversely related to bleeding events [39]. Evidence is still lacking in applying PFT to estimate bleeding risk. In our study, we found no difference in bleeding events between two groups, indicating only the safety of PFT-based intensified antiplatelet therapy.

Newly developed P2Y_12_ inhibitors prasugrel and ticagrelor are more potent and have a faster onset of action than clopidogrel, making them particularly attractive for patients undergoing PCI. In ACS patients exhibiting HPR on clopidogrel after PCI, both ticagrelor and prasugrel provide effective and sufficient platelet inhibition [40]. Studies have demonstrated that in patients with HPR, switching to prasugrel achieved adequate platelet inhibition and reduced thrombotic events [41, 42]. With increasing usage of newer P2Y_12_ inhibitors for coronary intervention, it may seem less necessary to concern about platelet resistance. However, there are still several issues unsettled. A proportion of patients treated with new P2Y_12_ inhibitors still display HPR, particularly during acute phase of myocardial infarction, putting forward an issue of how to deal with these non-responders. More importantly, new P2Y_12_ inhibitors carry an increased bleeding risk, so it would be challenging to tailor these agents with a balance between ischemic events and hemorrhagic complications. Platelet function testing may help sort out patients with benefit/risk ratio favorable for more potent anti-platelet therapy. As indicated in our analysis, the individualized approach based on PFT can improve net clinical benefit and should be considered to guide anti-platelet regime in future practice. Still, an appropriate defining of therapeutic window based on different methods of PFT needs further investigation to optimize intensified anti-platelet therapy.

It is noteworthy that our study included target vessel revascularization in the endpoint. The rationale of incorporating TVR in our analysis was that several studies have demonstrated a significant increase in revascularization rate in patients with HPR [43, 44]. More importantly, though still controversial, the notion that intensified antiplatelet therapy may have an inhibitory effect upon in-stent restenosis has been supported by some studies. For example, the DECLARE study series showed that cilostazol-based triple antiplatelet therapy could reduce angiographic restenosis and target lesion revascularization [45, 46]. Experimental research also indicated that the more potent ticagrelor could effectively inhibit neointima formation and potentially reduce restenosis [47]. Furthermore, the recent TRANSLATE-ACS study showed that switching clopidogrel to prasugrel led a significant decrease in unplanned revascularization rate [44]. In agreement with above findings, our pooled result also revealed association between intensified P2Y12 inhibition and reduction of TVR. However, since current level of evidence is still low, further study is needed to confirm whether more potent anti-platelet regime would decrease in-stent restenosis and incidence of TVR.

Our study has several limitations that should be taken into account. First, since there is no general agreement on standard definition of platelet resistance, studies included in our analysis used different definitions of platelet resistance or non-responsiveness. Defining HPR also varies according to the method of PFT, though there is an adequate correlation between different tests [48]. Since HPR cutoff values reported in many studies are associated with high negative predictive values and low positive predictive values, it may have an impact on the pooled results. Second, while most studies defined MACE as composite of cardiovascular death, myocardial infarction and definite/probable stent thrombosis, three studies included stroke/TIA in MACE as well. However, due to low incidence of stroke/TIA in these studies, the difference in the definition of MACE across studies is unlikely to affect our pooled results. Third, trials included in our study used different strategies to overcome HPR, so the extent of platelet inhibition is not same. Tailored therapy like doubling the dose of clopidogrel may still be insufficient to repress platelet activity. Forth, risk profile of patients included in our study was different. Patients in high risk group tend to have higher cardiovascular events, which may also affect the pooled results. What’s more, a small part of patients with low on-treatment reactivity were mixed in platelet function monitoring group in some studies, such as ARCTIC and ANTARCTIC trials. Such design may partly neutralize the benefit of tailored therapy, and have some affect in the pooled results.

## Conclusion

In conclusion, the present meta-analysis from RCTs suggests that in patients undergoing PCI who have HPR based on platelet function testing, intensified dual anti-platelet therapy reduces the incidence of thrombotic events without increasing the risk of major bleeding compared with standard clopidogrel therapy.

## Additional file 1

Additional file 1: Supplemental Material. (DOC 365 kb)

## Abbreviations

ACS: Acute coronary syndrome
CI: Confidence interval
HPR: High on-treatment platelet reactivity
MACE: Major adverse cardiovascular event
LD: Loading dose
MD: Maintenance dose
MI: Myocardial infarction
NSTEMI: Non-ST segment elevation myocardial infarction
RR: Risk ratio
PCI: Percutaneous coronary intervention
RCT: Randomized, controlled trial
ST: Stent thrombosis
STEMI: ST-segment elevation myocardial infarction
TVR: Target vessel revascularization
TIMI: Thrombolysis in Myocardial Infarction
