# Supplementary material for: Individualized dual antiplatelet therapy based on platelet function testing in patients undergoing percutaneous coronary intervention: a meta-analysis of randomized controlled trials
Source: BMC Cardiovasc Disord. 2017 Jun 15;17:157. doi: 10.1186/s12872-017-0582-6 (PMC5472866; doi:10.1186/s12872-017-0582-6)

S1 Cardiovascular death according to follow-up duration


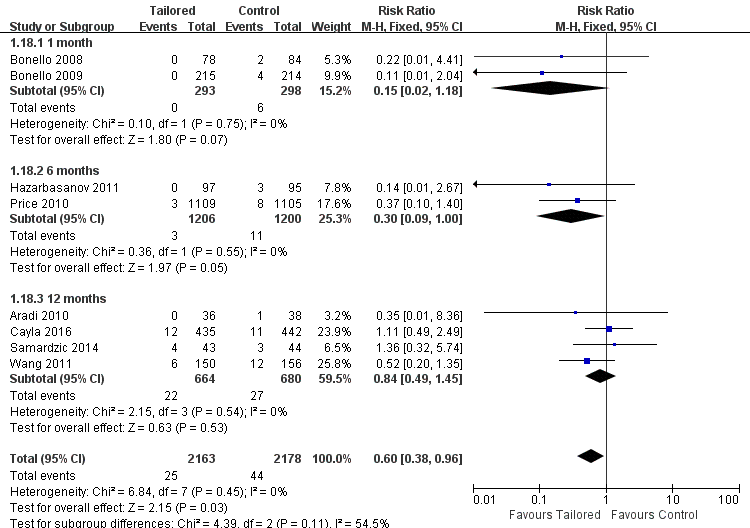


S2 Cardiovascular death according to intervention duration


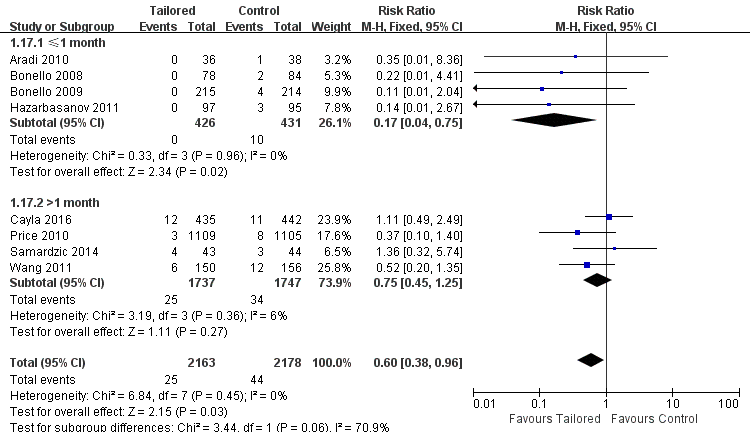


S3 Cardiovascular death according to method of platelet function testing


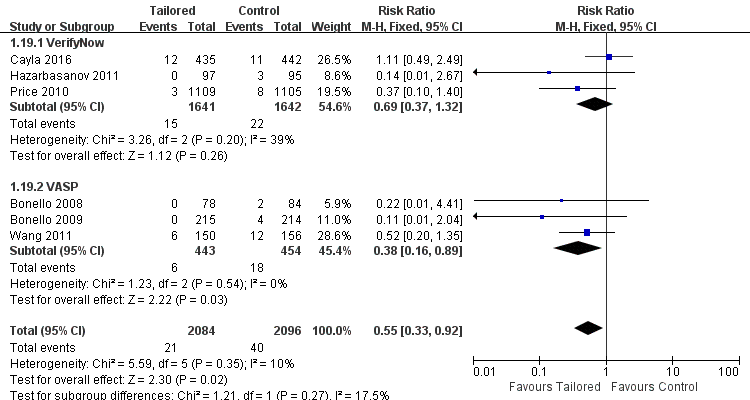


S4 Cardiovascular death according to strategies to overcome HPR


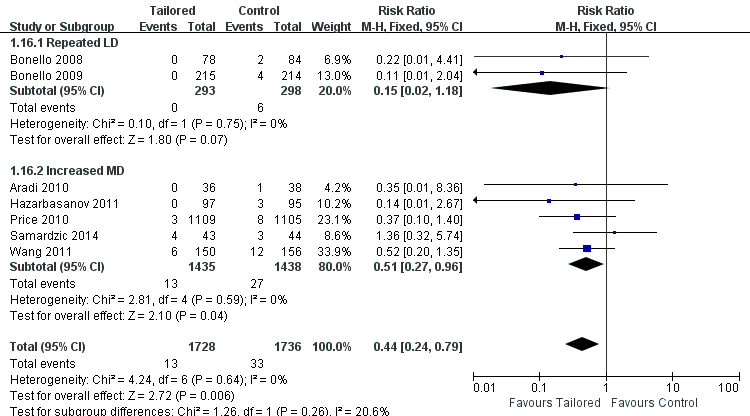


S5 MACE according to follow-up duration


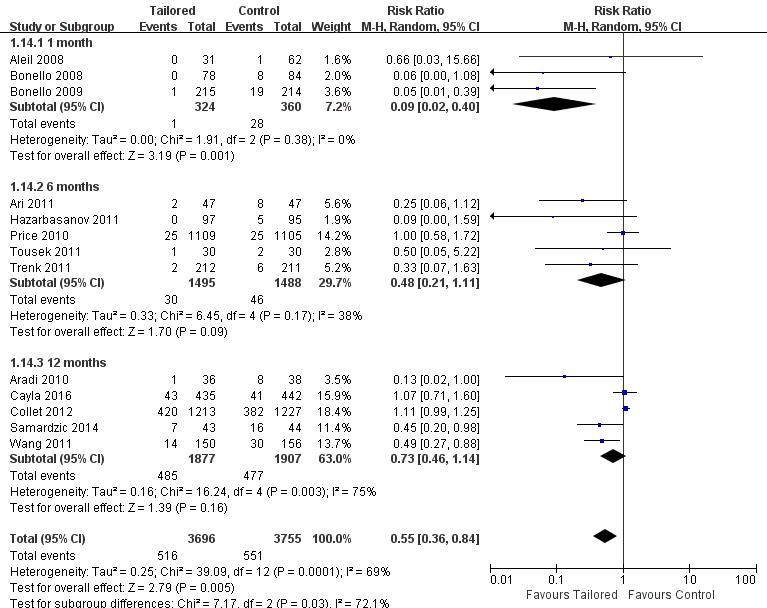


S6 MACE according to intervention duration


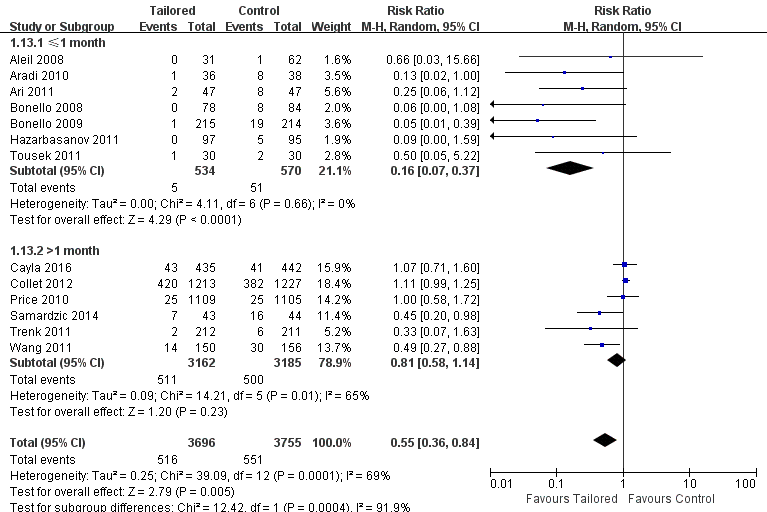


S7 MACE according to test of platelet function testing


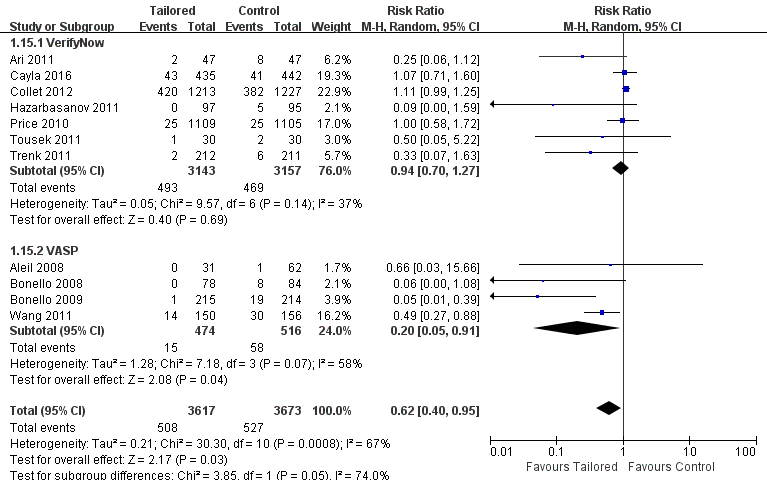


S8 MACE according to strategies to overcome HPR


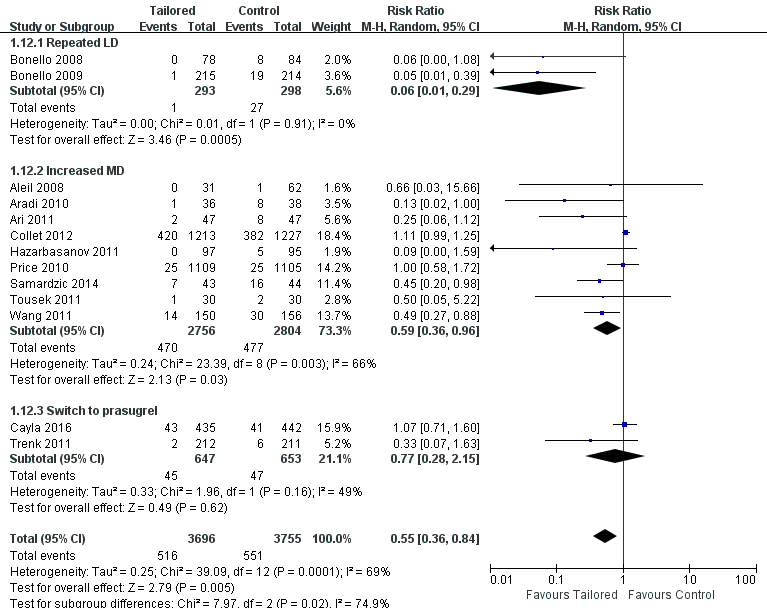


S9 Major bleeding according to follow-up duration


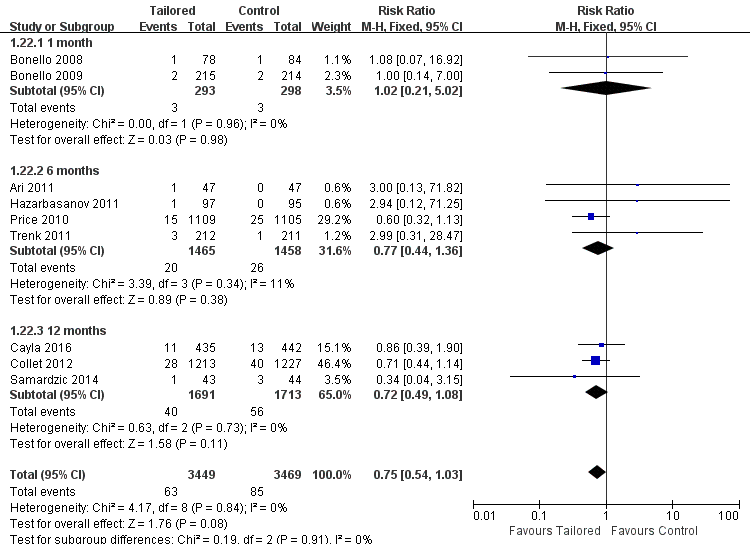


S10 Major bleeding according to intervention duration


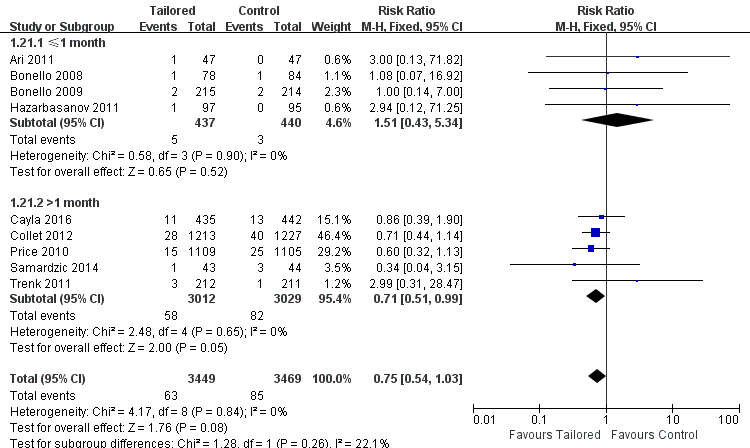


S11 Major bleeding according to method of platelet function testing


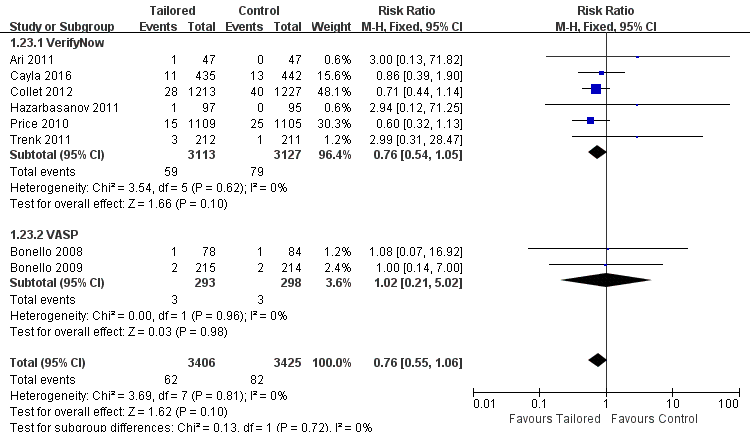


S12 Major bleeding according to strategies to overcome HPR


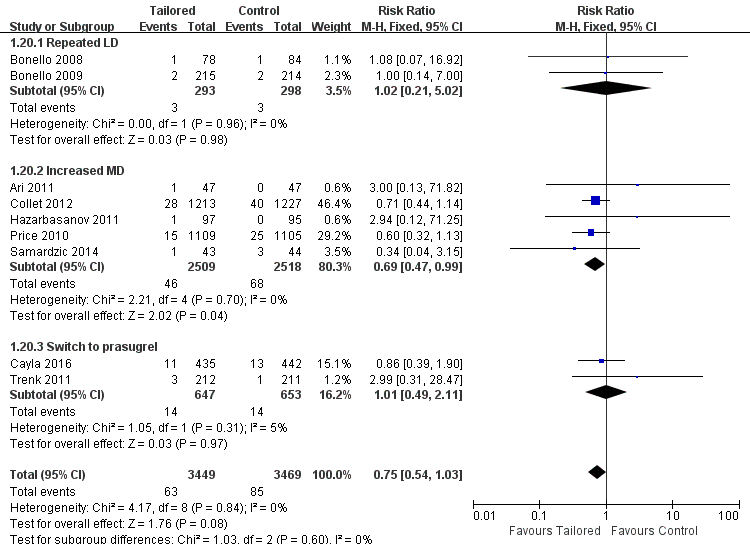


S13 Net clinical events according to follow-up duration


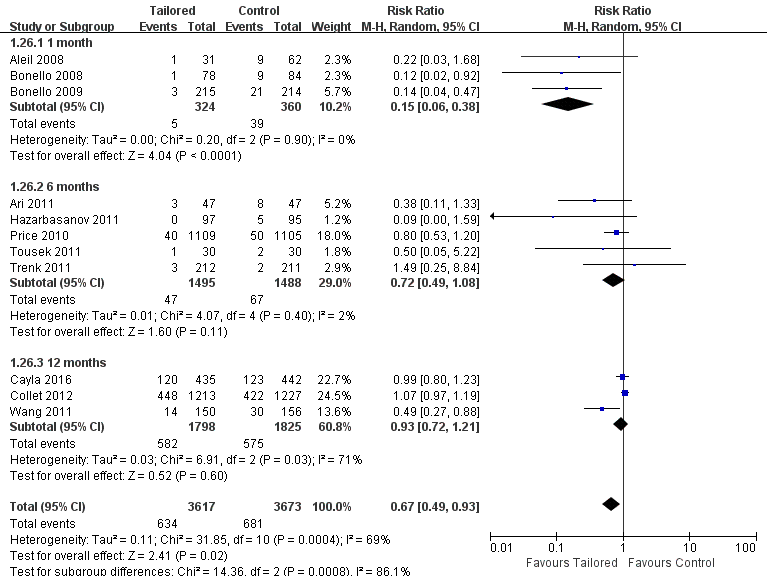


S14 Net clinical events according to intervention duration


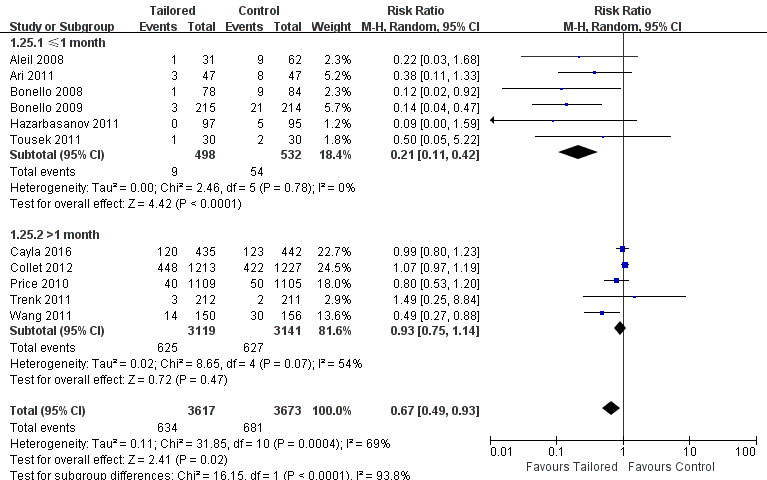


S15 Net clinical events according to method of platelet function testing


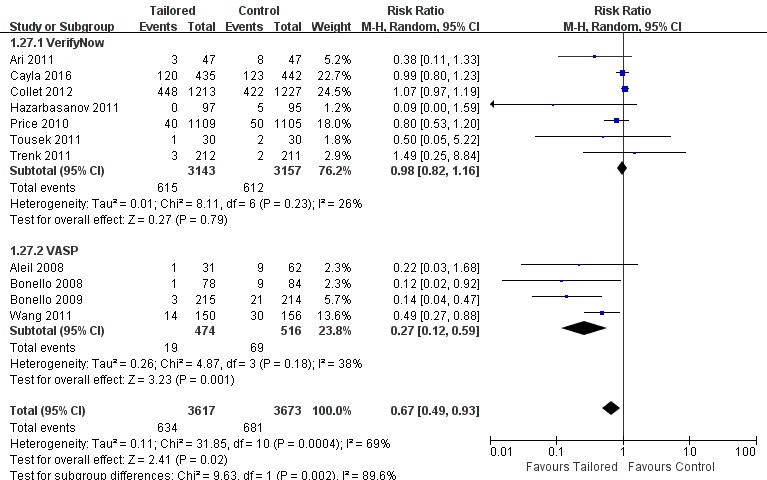


S16 Net clinical events according to strategies to overcome HPR


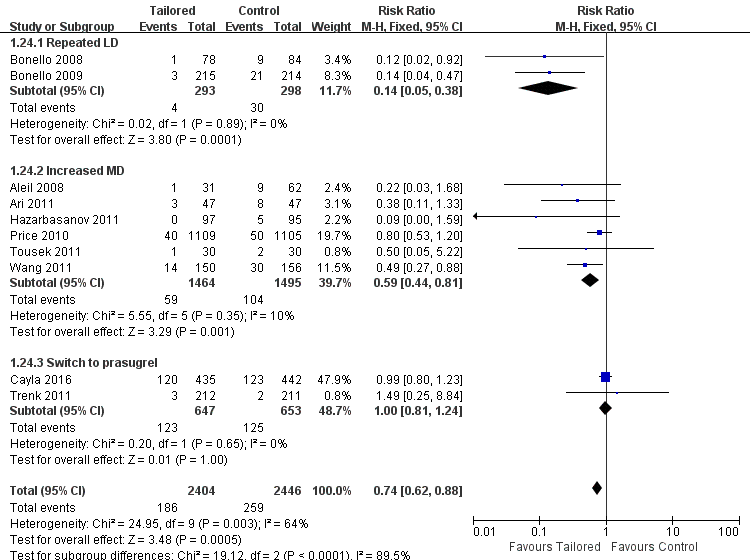


S17 MACE-SCAD


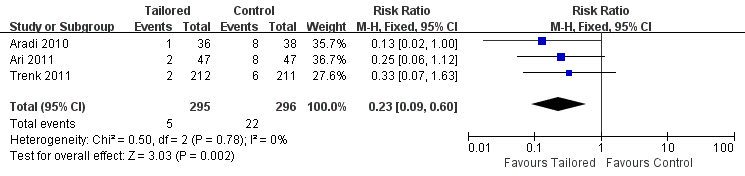

Supplement: Additional file 1: — Supplemental Material. (DOC 365 kb) [file 12872_2017_582_MOESM1_ESM.doc]
